# Severity of fatigue in people with rheumatoid arthritis, psoriatic arthritis and spondyloarthritis – Results of a cross-sectional study

**DOI:** 10.1371/journal.pone.0218831

**Published:** 2019-06-28

**Authors:** Trine Pilgaard, Lise Hagelund, Sandra Elkjær Stallknecht, Henrik Holm Jensen, Bente Appel Esbensen

**Affiliations:** 1 Pfizer Denmark A/S, Ballerup, Denmark; 2 Incentive, Holte, Denmark; 3 Copenhagen Center for Arthritis Research, Centre for Rheumatology and Spine Diseases, Centre for Head and Orthopaedics, Rigshospitalet, Glostrup, Denmark; 4 Department of Clinical Medicine, Faculty of Health and Medical Sciences, University of Copenhagen, Copenhagen, Denmark; Universita Campus Bio-Medico di Roma, ITALY

## Abstract

**Background:**

Despite improvements in treatment for rheumatoid arthritis (RA), psoriatic arthritis (PsA) and spondyloarthritis (axSpA), several key unmet needs remain, such as fatigue. The objective of this study was to describe the severity of fatigue, disease characteristics and socioeconomic factors in people with RA, PsA and axSpA.

**Methods:**

The study was designed as a cross-sectional survey collecting patient characteristics such as disease characteristics, socioeconomic factors and fatigue in people with RA, PsA and axSpA in Denmark. Respondents were consecutively recruited for the study over a six-month period in 2018 via routine visits to outpatient rheumatology clinics. Study nurses collected information on diagnosis, current disease-related treatment and disease activity from medical journals. People were invited to complete a questionnaire related to socioeconomic factors and containing the FACIT-Fatigue subscale. Descriptive statistics were analyzed using SAS.

**Results:**

We invited 633 people to participate, and 488 (77%) completed the questionnaire. Women constituted 62% of respondents, and the mean age was 53.5 years. Respondents had on average been diagnosed between 11 and 15 years ago. Overall, 79% had no changes to their disease-related treatment during the past year, and the average disease activity as indicated by DAS28 for RA and PsA was 2.48 and 2.36, respectively, and BASDAI for axSpA was 28.40. Fatigue was present in all three diagnoses (mean: 34.31). The mean fatigue score varied from respondents answering that they suffered from no or little fatigue (mean: 45.48) to extreme fatigue (mean: 10.11). Analyses demonstrated that the respondents were not considerably different from nonrespondents, and the study population is considered representative compared with Danish RA and axSpA patients in the Danish National Rheumatology Registry, the DANBIO database.

**Conclusion:**

We found that the majority of the study population were fatigued (61%). They had low disease activity and few disease-related treatment changes.

## Introduction

Fatigue is described as extreme tiredness, typically resulting from mental or physical exertion or illness [1]. However, an internationally accepted definition of fatigue does not exist [2]. One of the most commonly known illnesses to cause fatigue is inflammatory arthritis, and several studies have demonstrated that fatigue is a prevalent symptom in people with rheumatoid arthritis (RA), psoriatic arthritis (PsA) and spondyloarthritis (axSpA) [3–12]. Clinically important levels of fatigue are reported in 42% of the patients [13], while prevalence rates of 80% or more have also been reported in adults with RA [14–17], and it is perceived as a dominating problem with a greater impact on everyday life than pain [18].

In recent years, there has been an increased focus on fatigue and how fatigue correlates with other symptoms or conditions. According to a recent review of fatigue and RA, severe fatigue is common among individuals with RA and has a significant impact on quality of life [3]. In addition, RA-related factors (e.g., inflammation, pain) are associated with greater fatigue, but other factors, such as physical inactivity, sleep disturbance and depression, explain the major differences in fatigue [3]. Despite this recent increased focus on fatigue in arthritis, there is still a lack of research analyzing the severity of fatigue across diagnoses in inflammatory arthritis such as PsA and axSpA. Further, there is a lack of studies analyzing a potential correlation between fatigue and disease-specific characteristics and socioeconomic factors [3–12].

The prevalence of RA, PsA and axSpA varies globally. In Europe, the estimated prevalence is between 0.3% and 1% for RA [19], 0.001% and 0.42% for PsA [20], and 0.2% and 1.61% for axSpA [21]. Still, RA, PsA and axSpA are the most common types of inflammatory arthritis in the outpatient rheumatology clinics in Denmark. The estimated prevalence in the Danish population is 0.7% for RA [22], 0.1%-0.2% for PsA [23] and 0.3% for axSpA [24].

The objective of this study was to describe the severity of fatigue, disease characteristics and socioeconomic factors in people with RA, PsA and axSpA.

## Materials and methods

### Study population

This study was designed as a cross-sectional study of people with RA, PsA and axSpA currently enrolled at one of Rigshospitalet’s two outpatient clinics in Glostrup and Frederiksberg, Denmark. We aimed to recruit a representative sample of people with RA, PsA and axSpA, amounting to 527 (11% of patients with RA, PsA and axSpA enrolled in one of the two outpatient clinics). Inclusion criteria were people 18 and older with a confirmed diagnosis of RA, PsA or axSpA; who had been enrolled at one of the two clinics for more than four weeks; who were able to read, understand and write Danish; and who signed an informed consent to participate in the study. People were consecutively recruited between January and June 2018.

### Characteristics of respondents from medical journals

We developed a questionnaire, which was used by the study nurse to collect informed consent and characteristics from each respondent’s medical journal (S1 and S2 Files). Characteristics such as type of arthritis, age, gender, treatment (disease-modifying antirheumatic drug (DMARD) or biologic), treatment changes and disease activity score (Disease Activity Score-28 (DAS28), Bath Ankylosing Spondylitis Disease Activity Index (BASDAI) and/or Bath Ankylosing Spondylitis Functional Index (BASFI)) were obtained. For respondents eligible to participate, but not wishing to do so, information on diagnosis (RA, PsA, axSpA), gender, year of birth and reason for not wanting to participate in the study was collected if the respondent gave permission. This information was used for the dropout analysis.

### Patient-reported outcomes

We developed a patient questionnaire, which was used to collect patient demographics (S3 and S4 Files). It included outcomes such as years since diagnosis, marital status, education, employment status and household income. The questionnaire also included several other patient-reported outcomes (PROs), of which only the results of fatigue are reported in this study. The other results will be reported elsewhere. We used two scales to measure fatigue: the single visual analog scale (VAS) item from the Health Assessment Questionnaire (HAQ) [25] and the Functional Assessment of Chronic Illness Therapy (FACIT)-Fatigue scale [26], both validated to measure fatigue in patients with chronic illnesses, among others, in RA and PsA populations [10,27]. In the HAQ, people were asked: “How tired are you?” The scale ranges from 0 to 100, where 0 represents no fatigue and 100 represents severe fatigue. The FACIT-Fatigue scale is a 13-item questionnaire assessing self-reported fatigue. Originally developed as an addition to the Functional Assessment of Cancer Therapy measurement system, the FACIT-Fatigue scale developers have demonstrated that the fatigue subscale can stand alone as a brief, reliable and valid measuring tool. Items in the FACIT-Fatigue scale are scored 0–4 (five answer categories), and the total FACIT-Fatigue score ranges from 0 to 52 [28]. Based on this and to enable an analysis of severity of fatigue, we divided the responses into four grades of severity: respondents with no or little fatigue (composite score between 40 and 52), some fatigue (composite score between 27 and 39), quite a lot of fatigue (composite score between 14 and 26) and extreme fatigue (composite score between 0 and 13). If respondents scored 39 or lower on the FACIT-Fatigue scale, we categorized the respondents as suffering from fatigue. Thus, higher scores on the FACIT-Fatigue scale indicate less fatigue, while the opposite is true for the VAS where lower scores indicate less fatigue.

### Other measurements

This study is a part of a larger study “The impact of fatigue in patients with rheumatoid arthritis, psoriatic arthritis and spondyloarthritis in Denmark”. Besides the abovementioned, data on work productivity, health related quality of life, sleep and depression were collected, and will be reported elsewhere (in preparation).

### Data collection

The questionnaires were pilot-tested among five people with RA (not included in the analysis). The pilot was conducted in the same setting as the study cohort on a random day with people with RA, PsA and axSpA visiting the clinics for routine visits. The questionnaire was tested for comprehensibility, user friendliness and time consumption. As the primary part of the questionnaire was made up of validated scales, we did not test the comprehensibility of the specific scales but of the entire questionnaire, as well as the flow in the questionnaire. In general, the pilots answered within an expected time frame and found the questionnaire acceptable and understandable.

People with RA, PsA or axSpA were approached by a study nurse in each clinic and enrolled in the study based on informed consent. The study nurse collected characteristics via a medical journal while the person completed a self-reported questionnaire on an iPad. If the respondent preferred to complete the questionnaire at home, the respondent was either given a link to the online questionnaire or a paper version of the questionnaire and a stamped, addressed envelope and was asked to return the questionnaire by ordinary mail. Study participation was encouraged by the study nurse for those people who agreed to participate in the survey but for some reason did not answer the questionnaire. The study was conducted with approval from the Danish Data Protection Agency (j.nr. 2012-41-1199) and approval from the Ethics Committee of the Capital Region of Denmark (Protocol 17021125). The Danish Medicines Agency was informed about the execution of the study, as per current Danish guidelines (case no. 2017071026).

### Database and statistical analysis

The questionnaires were both set up in SurveyXact, an online questionnaire collection program developed by Rambøll. The answers of those respondents choosing to complete the questionnaire on paper were entered manually into SurveyXact. Statistical analyses were conducted in SAS and Microsoft Excel. Where applicable, we applied analysis of variance and Pearson’s chi-square test to test for significant differences between groups.

## Results

**Fig 1:**
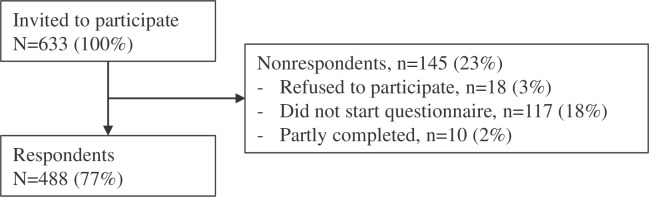
Flow chart.

We invited 633 people to complete the questionnaire, and of those, 488 completed it (77% response rate). Of the 145 (23%) who did not complete the questionnaire, 18 (3%) refused to participate, 117 (18%) agreed but never started the questionnaire, and 10 (2%) partly completed the questionnaire (Fig 1).

### Study population

In total, 488 respondents completed the questionnaire (450 electronically and 38 on paper). Of the 488, 60% had a diagnosis of RA, 17% a diagnosis of PsA and 23% a diagnosis of axSpA (Table 1). Of all respondents, 62% were women; however, the distribution across the three diagnoses was uneven, with 79% of the RA respondents being women while 46% and 30% of the PsA and axSpA respondents were women, respectively. The youngest population was found among the axSpA respondents (mean age: 43.6), with 35% of the axSpA respondents being between 18 and 39 years of age, compared with 13% and 16% for RA and PsA, respectively. The oldest population was found among the RA respondents (mean age: 57.4), with 49% of RA respondents being above 60 years of age. The majority of the respondents did not change treatment within the past 12 months (79%), a number that is very consistent across the three diagnoses (RA: 81%; PsA: 79%; axSpA: 73%). The current treatment varied across diagnoses, but on average, 39% were receiving biosimilar DMARD (bsDMARD), 18% conventional synthetic DMARD (csDMARD) and 42% both (1% no current treatment). The average disease activity as indicated by DAS28 for RA and PsA was 2.48 (standard deviation (SD): 1.11) and 2.36 (SD: 0.99), respectively, and BASDAI for axSpA was 28.40 (SD: 23.78). BASFI was 30.93 (SD: 22.93) for PsA and 27.65 (SD: 23.93) for axSpA.

**Table 1:** Characteristics from medical journals.

	All	RA	PsA	axSpA
	N (%)	n (%)	n (%)	n (%)
All	488 (100%)	293 (60%)	85 (17%)	110 (23%)
Sex				
Male	185 (38%)	62 (21%)	46 (54%)	77 (70%)
Female	303 (62%)	231 (79%)	39 (46%)	33 (30%)
Age				
18–39 years	91 (19%)	38 (13%)	14 (16%)	39 (35%)
40–59 years	221 (45%)	111 (38%)	46 (54%)	64 (58%)
60+ years	176 (36%)	144 (49%)	25 (29%)	7 (6%)
Age in years, mean (SD)	*53*.*5 (14*.*5)*	*57*.*4 (14)*	*52*.*6 (13*.*2)*	*43*.*6 (11*.*9)*
Treatment change in the past 12 months				
0	385 (79%)	238 (81%)	67 (79%)	80 (73%)
1	92 (19%)	46 (16%)	17 (20%)	29 (26%)
2 or more	11 (2%)	9 (3%)	1 (1%)	1 (1%)
Current treatment				
bsDMARD	191 (39%)	69 (24%)	33 (39%)	89 (81%)
csDMARD	90 (18%)	68 (23%)	22 (26%)	0 (0%)
bsDMARD and csDMARD	204 (42%)	155 (53%)	29 (34%)	20 (18%)
No current treatment	3 (1%)	1 (0%)	1 (1%)	1 (1%)
Disease activity status				
DAS28, mean (SD)	*2*.*45 (1*.*08)*	*2*.*48 (1*.*11)*	*2*.*36 (0*.*99)*	*2*.*35 (0*.*9)*
BASDAI, mean (SD)	*30*.*59 (23*.*56)*	*NA*	*33*.*54 (23*.*07)*	*28*.*40 (23*.*78)*
BASFI, mean (SD)	*29*.*05 (23*.*51)*	*NA*	*30*.*93 (22*.*93)*	*27*.*65 (23*.*93)*

The sum of percentages does not reach 100% due to rounding errors.

### Patient-reported socioeconomic characteristics

Among the newly diagnosed (0–5 years), the highest share of respondents was among those with axSpA (30%) (Table 2). More than half of the respondents (64%) were married/living together with a partner (with or without children) and there was little variation between the diagnoses. Almost half of the respondents (46%) had a bachelor’s degree or higher, and the majority of the population belonged to the active workforce (58%). Finally, more than half had a household income above 400,000 DKK.

**Table 2:** Socioeconomic characteristics (patient-reported).

	All	RA	PsA	axSpA
	N (%)	n (%)	n (%)	n (%)
All	488 (100%)	293 (60%)	85 (17%)	110 (23%)
Years since diagnosis				
0–5 years	105 (22%)	57 (19%)	15 (18%)	33 (30%)
6–10 years	105 (22%)	62 (21%)	24 (28%)	19 (17%)
11–15 years	110 (23%)	71 (24%)	17 (20%)	22 (20%)
16–20 years	70 (14%)	39 (13%)	14 (16%)	17 (15%)
More than 20 years	98 (20%)	64 (22%)	15 (18%)	19 (17%)
Type of household				
Married/living together^a^	312 (64%)	185 (63%)	56 (66%)	71 (65%)
Single^b^	162 (33%)	103 (35%)	25 (29%)	34 (31%)
Living at home with parents or house sharing with other adults	13 (3%)	4 (1%)	4 (5%)	5 (5%)
Highest obtained education				
Elementary school or high school	99 (20%)	58 (20%)	20 (24%)	22 (20%)
Secondary or short-cycle tertiary	166 (34%)	94 (32%)	33 (39%)	39 (35%)
Bachelor’s or higher	222 (46%)	141 (48%)	32 (38%)	49 (45%)
Employment status				
Employed^c^	281 (58%)	148 (51%)	48 (57%)	85 (77%)
Unemployed	19 (4%)	8 (3%)	7 (8%)	4 (4%)
Retired^d^	123 (25%)	100 (34%)	17 (20%)	6 (5%)
Homemaker, student or other	62 (13%)	35 (12%)	12 (14%)	15 (14%)
Total household income				
Below 400,000 DKK	183 (38%)	112 (39%)	28 (33%)	43 (39%)
400,000 DKK or more	250 (52%)	140 (48%)	48 (56%)	62 (56%)
Refused to answer	51 (11%)	37 (13%)	9 (11%)	5 (5%)

The sum of percentages does not reach 100% due to rounding errors. Some socioeconomic information was not available for all respondents.

^a^Married/living together with or without children.

^b^Single with or without children.

^c^Employed full time, part time or flexible hours.

^d^Age-related retirement or health-related early retirement because of arthritis or other reason.

### Nonrespondents

When analyzing the nonrespondents according to characteristics, it became evident that nonrespondents were distributed evenly across the diagnoses compared with respondents (Table 3). However, there were significantly more men in the nonrespondent group (50%) than the respondent group (38%) (p = 0.0075). The average age of the nonrespondents was 50 years vs. 53 in the respondent group (p = 0.0489). The percentage of nonrespondents was higher in the youngest age group (18–39) than in the respondent group (28% vs. 19%), and, finally, there was no difference with regard to treatment changes.

**Table 3:** Analysis of nonrespondents.

	Nonrespondents	Study population	P-value chi-square test
	n (%)	n (%)	
All	145 (100%)	488 (100%)	
Diagnosis			0.3446
RA	79 (54%)	293 (60%)	
PsA	25 (17%)	85 (17%)	
axSpA	41 (28%)	110 (23%)	
Sex			0.0075
Male	73 (50%)	185 (38%)	
Female	72 (50%)	303 (62%)	
Age			0.0489
18–39 years	40 (28%)	91 (19%)	
40–59 years	63 (43%)	221 (45%)	
60+ years	42 (29%)	176 (36%)	
Mean age	*50*	*53*	
Treatment change in the past 12 months^a^			0.3861
0	93 (73%)	385 (79%)	
1	30 (24%)	92 (19%)	
2 or more	4 (3%)	11 (2%)	

^a^Out of 145 nonrespondents, 18 did not register a treatment change within the past 12 months (n = 127).

### Representativity

In Denmark, data on people with RA and axSpA currently receiving DMARDs is routinely collected at scheduled visits to rheumatology centers in the Danish National Rheumatology Registry, the DANBIO database. However, the DANBIO database do not include information on people with PsA. The data collected includes age and gender, type of treatment, and treatment changes registered by the nurse or physician as well as patient-reported data (HAQ scale) [25,29]. This data allows us to analyze whether the study cohort is representative of both the Glostrup/Frederiksberg cohort and the full Danish RA and axSpA population.

In 2017, there were 4,788 people with RA, PsA and axSpA enrolled at the Glostrup and Frederiksberg outpatient rheumatology clinics [30]. We targeted 527 people with RA, PsA and axSpA, and of those, 488 (93% of target) completed the questionnaire, with 96% of RA, 83% of PsA and 91% of axSpA participating in the study (Table 4).

**Table 4:** Respondents.

	RA	PsA	axSpA	Total
Number of people enrolled, in total	2,764	924	1,100	4,788
Glostrup	1,600	557	656	2,813
Frederiksberg	1,164	367	444	1,975
Target	304	102	121	527
Invited/eligible people	NA	NA	NA	633
Respondents	293	85	110	488
Percentage of target	96%	83%	91%	93%

When comparing the study cohort with the Glostrup and Frederiksberg cohort from DANBIO 2017, the RA respondents are slightly younger than the average Glostrup and Frederiksberg cohort from 2017 (56/60 vs. 61/60 for RA and 45/42 vs. 45/41 for axSpA, respectively) (Table 5). Further, there are slightly more RA women in the study cohort than in the cohort from Glostrup and Frederiksberg (78%/80% vs. 77%/76%) and slightly fewer axSpA women in the study cohort than in the cohort from Glostrup and Frederiksberg (29%/31% vs. 35%/34%) [31]. When comparing the study cohort with the full Danish RA and axSpA population from DANBIO 2017, the RA and axSpA respondents are slightly younger than the DANBIO cohort average (57 vs. 63 for RA and 44 vs. 45 for axSpA, respectively). Further, there are more RA women and fewer axSpA women in the study cohort than in the DANBIO cohort (79% vs. 71% for RA and 30% vs. 40% for axSpA, respectively) [31].

**Table 5:** Representability compared with DANBIO data.

	Study population (Glostrup)		Study population (Frederiksberg)		Study population (all)		DANBIO (Glostrup)		DANBIO (Frederiksberg)		DANBIO (all Denmark)	
	RA	axSpA	RA	axSpA	RA	axSpA	RA	axSpA	RA	axSpA	RA	axSpA
Women (%)	78%	29%	80%	31%	79%	30%	77%	35%	76%	34%	71%	40%
Mean age	56	45	60	42	57	44	61	45	60	41	63	45

### Fatigue

The mean fatigue scores on the VAS were 42.16 (SD: 29.95), 49.69 (SD: 29.18) and 45.01 (SD: 29.57) for RA, PsA and axSpA, respectively. The mean VAS fatigue scores did not vary statistically significantly among the three diagnoses (p = 0.11). On the FACIT-Fatigue scale, the mean fatigue score was 34.31 (SD: 6.08), and fatigue was present in all three diagnoses (Table 6). The mean fatigue scores across diagnoses was not significant (p = 0.078). The mean fatigue score was 34.86 (SD: 11.04) among RA respondents, 31.83 (SD: 10.89) among PsA respondents and 34.77 (SD: 11.44) among axSpA respondents. The mean fatigue score varied among respondents, indicating that they suffered from no or a little fatigue (mean: 45.48; SD: 3.62) to extreme fatigue (mean: 10.11; SD: 3.61). In total, 61% of the respondents expressed fatigue (some, quite a lot or extreme).

**Table 6:** Fatigue measured by FACIT-Fatigue score.

Mean (SD)		All	RA	PsA	axSpA
		N (%)	n (%)	n (%)	n (%)
Number		487 (100%)	292 (100%)	85 (100%)	110 (100%)
Mean fatigue (SD)		34.31 (6.08)	34.86 (11.04)	31.83 (10.89)	34.77 (11.44)
Severity of fatigue					
No or little fatigue^a^	45.48 (3.62)	189 (39%)	120 (41%)	23 (27%)	46 (42%)
Some fatigue^b^	33.05 (3.69)	171 (35%)	99 (34%)	39 (46%)	33 (30%)
Quite a lot of fatigue^c^	20.91 (3.57)	109 (22%)	62 (21%)	19 (22%)	28 (25%)
Extreme fatigue^d^	10.11 (3.61)	18 (4%)	11 (4%)	4 (5%)	3 (3%)

Higher fatigue scores indicate less fatigue.

^a^FACIT-Fatigue score: 40–52.

^b^FACIT-Fatigue score: 27–39.

^c^FACIT-Fatigue score: 14–26.

^d^FACIT-Fatigue score: 0–13.

## Discussion

In this study, 488 respondents (77% response rate) completed the questionnaire, and fatigue was frequent in all three diagnoses (RA, PsA and axSpA). Women constituted 62% of respondents, and the mean age was 53.5 years, which is in accordance with other studies that have found that more fatigue has been reported in association with advancing age [32,33] and female sex [32–35]. We also found that the study population had low disease activity and was relatively stable, with 79% reporting no changes to their disease-related treatment during the past year.

Despite this, the study population suffered from fatigue. We measured fatigue in two ways. On the VAS, we found a mean fatigue score of 42.16 (SD: 29.95), 49.69 (SD: 29.18) and 45.01 (SD: 29.57) for RA, PsA and axSpA, respectively. Our findings are consistent with mean fatigue scores previously documented in a cross-sectional Norwegian study (mean fatigue score on the VAS: 39.9, 43.6 and 43.6 for RA, PsA and axSpA, respectively) [36]. We also used the FACIT-Fatigue instrument to evaluate fatigue. Fatigue was present in all three diagnoses, with a mean FACIT-Fatigue score of 34.31 (SD: 6.08) across diagnoses, which was lower than previously reported among a large representative sample (N = 2,426) of the average German population (mean: 43.5; SD: 8.3) [37] and a large sample (N = 1,010) of the general U.S. population (mean: 43.6; SD: 9.4) [38]. Among the studies we identified measuring fatigue by the FACIT-Fatigue score in people with RA, the score ranged from 34.6 to 42 [39–41] (the fatigue score of 42 was found among RA patients with low disease activity), which is somewhat higher than the mean fatigue score identified in the current study (mean: 34.86; SD 11.04). The mean FACIT-Fatigue score among PsA patients was slightly lower (mean: 31.83; SD: 10.89) than previously reported in a study of Canadian people with PsA (mean: 35.8) [10]. To date, no studies have, to our knowledge, measured fatigue by the FACIT-Fatigue score among axSpA patients. In total, 61% (RA: 59%; PsA: 73; axSpA: 58%) of the respondents expressed that fatigue was a symptom they were affected by to some extent. This is in line with the prevalence of fatigue previously reported among patients with RA, found to vary from 40% to 80% [3,42,43], and the prevalence among PsA patients, found to vary from 44.7% to 78.2% [11,12]. The prevalence of fatigue by FACIT-Fatigue score among axSpA patients has not been estimated in any previous research.

The study population from both disease-specific and socioeconomic perspectives was a well-functioning group of people. Also, overall, the population belonged to the active workforce (58%). This is noteworthy because a recently published study that aimed to determine factors associated with work productivity found a strong association of fatigue with all work measures as well as with daily activity impairment [44]. This emphasizes the need for focusing on increased knowledge about and focus on how to reduce fatigue, which may help optimize work status in people with inflammatory arthritis.

To date, the FACIT-Fatigue scale has been used to measure progress in fatigue in both cross-sectional and longitudinal studies. These studies have reported a minimal important difference of 3–8.3 on the FACIT-Fatigue scale from 0 to 52 [45].

### Strengths and limitations

Our study has several strengths. One of the main strengths of the study is the data source for the patient characteristics, which were recorded by trained study nurses from the respondents’ medical journals. This method excludes potential recall biases seen in surveys exclusively based on patient-reported data. Another strength of the study is the recruitment method, which has ensured a high response rate. Similarly, the dropout analysis showed that the respondents were not considerably different from the nonrespondents. Finally, our study is considered representative of the Danish RA, PsA and axSpA populations.

We acknowledge, however, that the study has some limitations. The study is cross-sectional and can therefore not provide any conclusions concerning causality. It can generate only hypotheses, which can be further analyzed in future prospectively conducted studies. Despite a good representation in the study cohort of both genders and age groups, the study cohort is slightly older and consists of more women than the group of nonrespondents. The same tendency is seen in other surveys [46]. The study is further limited by its geographical scope, which is limited to two clinics in the Capital Region of Denmark. The study cohort is slightly younger than the average Danish DANBIO population, and there were slightly more RA women and fewer axSpA respondents in the study cohort. But as the data collection method did not differ from that of DANBIO, we assume that the explanation is due to chance, as the data collection period did not last a full year but covered only six months.

Scales differ in their focus, some measuring severity only and others duration and impact on a range of functions as well as different aspects of fatigue. In this study, we focused on measuring the severity of fatigue using both VAS and FACIT-Fatigue scores, which measured one aspect of fatigue (unidimensional) and not the multidimensional aspects of fatigue, which can be viewed as a limitation.

## Conclusion

We found that the majority of the study population was fatigued. The study cohort represents a stable group with low disease activity, few disease-related treatment changes and strong attachment to the labor market. The study cohort is considered representative, and the results warrant further analyses of how fatigue correlates with other PROs and how it differs across the diagnoses.

## Supporting information

S1 File Nurse questionnaire in Danish (original language). (DOCX)

S2 File Nurse questionnaire in English. (DOCX)

S3 File Patient questionnaire in Danish (original language). (DOCX)

S4 File Patient questionnaire in English. (DOCX)
