# Supplementary material for: Severity of fatigue in people with rheumatoid arthritis, psoriatic arthritis and spondyloarthritis – Results of a cross-sectional study
Source: PLoS One. 2019 Jun 28;14(6):e0218831. doi: 10.1371/journal.pone.0218831 (PMC6599141; doi:10.1371/journal.pone.0218831)
Supplement: S1 File — (DOCX) [file pone.0218831.s001.docx]

# Undersøgelsen om gigt og træthed

1. Deltagelse i undersøgelsen

**A1 Vil patienten deltage?**

❑ Ja

❑ Nej (Hvis nej, gå videre til spørgsmål C1)

1. Patienter der ønsker at deltage

**B1 Hvilken sygdom er patienten diagnosticeret med?**

❑ Reumatoid artrit (RA)

❑ Psoriasisartrit (PsA)

❑ Spondylartrit (SpA)

**B2 Hvad er patientens køn?**

❑ Mand

❑ Kvinde

**B3 Hvilket år er patienten født?**

___ ___ ___ ___ *(Angiv årstal ÅÅÅÅ)*

**B4 Hvilken gigtbehandling er patienten i nu?***(Angiv kun DMARD og/eller biologisk behandling)*

|  |
| --- |

❑ Ingen

**B5 Hvor mange gange har patienten ændret behandling inden for de sidste 12 måneder?**

❑ 0

❑ 1

❑ 2 eller flere

**B6 Hvad er patientens sygdomsstatus?***Skriv de relevante mål med information fra DANBIO*

DAS28: _________________________

BASDAI: ________________________

BASFI: __________________________

1. Patienter der ikke ønsker at deltage

**C1 Hvis det er muligt, så få patientens accept til at notere svar på nedenstående spørgsmål.**

**C2 Hvilken sygdom er patienten diagnosticeret med?**

❑ Reumatoid artrit (RA)

❑ Psoriasisartrit (PsA)

❑ Spondylartrit (SpA)

**C3 Hvad er patientens køn?**

❑ Mand

❑ Kvinde

**C4 Hvilket år er patienten født?**

___ ___ ___ ___ *(Angiv årstal ÅÅÅÅ)*

**C5 Hvorfor ønsker patienten ikke at deltage?**

|  |
| --- |
