# Supplementary material for: Severity of fatigue in people with rheumatoid arthritis, psoriatic arthritis and spondyloarthritis – Results of a cross-sectional study
Source: PLoS One. 2019 Jun 28;14(6):e0218831. doi: 10.1371/journal.pone.0218831 (PMC6599141; doi:10.1371/journal.pone.0218831)
Supplement: S2 File — (DOCX) [file pone.0218831.s002.docx]

# The study on arthritis and fatigue

1. Participation in the study

**A1 Will the patient participate?**

❑ Yes

❑ No (If no, go to question C1)

1. Patients who wish to participate

**B1 Which disease is the patient diagnosed with?**

❑ Rheumatoid arthritis (RA)

❑ Psoriatic arthritis (PsA)

❑ Spondyloarthritis (axSpA)

**B2 What is the patient’s gender?**

❑ Man

❑ Woman

**B3 In what year was the patient born?**

___ ___ ___ ___ *(Enter year YYYY)*

**B4 Which treatment for arthritis is the patient currently receiving?***(Enter only DMARD and/or biologic treatment)*

|  |
| --- |

❑ None

**B5 How many times has the patient changed treatment within the past 12 months?**

❑ 0

❑ 1

❑ 2 or more

**B6 What is the patient’s disease activity status?***Write the relevant measure with information from the DANBIO database*

DAS28: _________________________

BASDAI: ________________________

BASFI: __________________________

1. Patients who do not wish to participate

**C1 If possible, get the patient’s acceptance to record answers to the questions below.**

**C2 Which disease is the patient diagnosed with?**

❑ Rheumatoid arthritis (RA)

❑ Psoriatic arthritis (PsA)

❑ Spondyloarthritis (axSpA)

**C3 What is the patient’s gender?**

❑ Man

❑ Woman
 **C4 In what year was the patient born?**

___ ___ ___ ___ *(Enter year YYYY)*

**C5 Why does the patient not want to participate?**

|  |
| --- |
