# Supplementary material for: Severity of fatigue in people with rheumatoid arthritis, psoriatic arthritis and spondyloarthritis – Results of a cross-sectional study
Source: PLoS One. 2019 Jun 28;14(6):e0218831. doi: 10.1371/journal.pone.0218831 (PMC6599141; doi:10.1371/journal.pone.0218831)
Supplement: S3 File — (DOCX) [file pone.0218831.s003.docx]

# Tak, fordi du vil deltage

i undersøgelsen om gigt og træthed, som vi gennemfører på Videnscenter for Reumatologi og Rygsygdomme i efteråret/vinteren 2017.

| Velkommen til spørgeskemaet Vi er glade for, at du ønsker at deltage.  Spørgeskemaet indeholder spørgsmål om, hvordan du har det i hverdagen med din gigtsygdom herunder smerter, træthed og søvn, oplevet livskvalitet samt arbejdsevne. Du vil måske opleve, at nogle spørgsmål minder om hinanden. Det er vigtigt, at du svarer på alle spørgsmålene alligevel.  Det tager cirka 20 minutter at besvare spørgeskemaet. Alle oplysninger der indsamles i forbindelse med spørgeskemaet vil blive behandlet fortroligt. |
| --- |

Skriv det firecifrede løbenummer

___ ___ ___ ___

| 1. Baggrund og uddannelse   De første spørgsmål i spørgeskemaet relaterer sig til din baggrund og uddannelse. |
| --- |

A1 Hvilket år er du født?
*Skriv årstallet med fire tal: ÅÅÅÅ*

___ ___ ___ ___

**A2 For hvor mange år siden blev du diagnosticeret med gigt?**
*Sæt ét kryds*

❑ 0-5 år

❑ 6-10 år

❑ 11-15 år

❑ 16-20 år

❑ Mere end 20 år

A3 I hvilken type husstand bor du?
*Sæt ét kryds*

❑ Gift/samboende med børn

❑ Gift/samboende uden børn

❑ Alene med børn

❑ Alene uden børn

❑ Hjemmeboende hos forældre

❑ Bofællesskab med andre voksne

A4 Hvad er dit højeste niveau af uddannelse?
*Sæt ét kryds*

❑ Folkeskole

❑ Gymnasial uddannelse

❑ Erhvervsuddannelse

❑ Kort videregående uddannelse

❑ Mellemlang videregående uddannelse

❑ Lang videregående uddannelse

**A5 Hvad er din nuværende beskæftigelsessituation (hovedbeskæftigelse)?**

*Sæt ét kryds*

❑ Fuldtidsansat

❑ Deltidsansat

❑ Fleksjob

❑ Studerende

❑ Hjemmegående

❑ Arbejdsledig

❑ Aldersbetinget pension

❑ Helbredsbetinget førtidspension pga. leddegigt

❑ Helbredsbetinget førtidspension af andre årsager

❑ Andet. Uddyb gerne:

**A6 Hvad er din husstands årlige indkomst før skat?**
*Sæt ét kryds*

❑ Under 200.000 kr.

❑ 200.000 kr. - 399.999 kr.

❑ 400.000 kr. - 599.999 kr.

❑ 600.000 kr. eller mere

❑ Ønsker ikke at svare

| 1. Velbefindende   Spørgsmålene på de næste sider handler om dit fysiske, sociale, følelsesmæssige og funktionelle velbefindende. Spørgsmålene er formuleret som udsagn. Du bedes sætte en ring omkring et tal ud for hvert udsagn, så svaret passer bedst mulig med, hvordan du har haft det de seneste 7 dage. |
| --- |

| Nedenfor er anført en række udsagn, som andre mennesker med din sygdom har sagt, er vigtige. **Ved at vælge ét tal i hver linje bedes du angive dit svar, sådan at det passer på de seneste 7 dage.** | | | | | | |
| --- | --- | --- | --- | --- | --- | --- |
| **FYSISK VELBEFINDENDE** | | **Slet ikke** | **En lille smule** | **I nogen grad** | **En hel del** | **Meget** |
|  | |  |  |  |  |  |
| Jeg mangler energi………………………………................. | | 0 | 1 | 2 | 3 | 4 |
| Jeg har kvalme…………………………………………….. | | 0 | 1 | 2 | 3 | 4 |
| På grund af min fysiske tilstand har jeg svært ved at  opfylde min families/mine nærmestes behov……………… | | 0 | 1 | 2 | 3 | 4 |
| Jeg har smerter…………………………………………….. | | 0 | 1 | 2 | 3 | 4 |
| Jeg er generet af bivirkninger af behandlingen……………. | | 0 | 1 | 2 | 3 | 4 |
| Jeg føler mig syg………………………………….............. | | 0 | 1 | 2 | 3 | 4 |
| Jeg er tvunget til at være sengeliggende noget af tiden…... | | 0 | 1 | 2 | 3 | 4 |
|  | |  |  |  |  |  |
| **SOCIALT/FAMILIEMÆSSIGT VELBEFINDENDE** | | **Slet ikke** | **En lille smule** | **I nogen grad** | **En hel del** | **Meget** |
|  | |  |  |  |  |  |
| Jeg føler mig tæt knyttet til mine venner……...….............. | | 0 | 1 | 2 | 3 | 4 |
| Jeg får følelsesmæssig støtte fra min familie/mine  nærmeste …………………………………………………. | | 0 | 1 | 2 | 3 | 4 |
| Jeg får støtte fra mine venner …………………………….. | | 0 | 1 | 2 | 3 | 4 |
| Min familie/mine nærmeste har accepteret min sygdom… | | 0 | 1 | 2 | 3 | 4 |
| Jeg er tilfreds med den måde, vi taler om sygdommen på  i familien/blandt mine nærmeste ………………………… | | 0 | 1 | 2 | 3 | 4 |
| Jeg føler mig tæt knyttet til min partner (eller den person,  der er min bedste støtte)………....……………………….. | | 0 | 1 | 2 | 3 | 4 |
| Uanset om du er seksuelt aktiv eller ej, bedes du venligst besvare følgende spørgsmål - Hvis du ikke har lyst til at besvare spørgsmålet, bedes du sætte kryds i boksen  og gå videre til næste udsagn*.* | |  |  |  |  |  |
| Jeg er tilfreds med mit sexliv……………………………... | | 0 | 1 | 2 | 3 | 4 |
| **Ved at vælge ét tal i hver linje bedes du angive dit svar, sådan at det passer på de seneste 7 dage.** | | | | | | |
| **FØLELSESMÆSSIGT VELBEFINDENDE** | | **Slet ikke** | **En lille smule** | **I nogen grad** | **En hel del** | **Meget** |
|  | |  |  |  |  |  |
| Jeg er ked af det……………………………………………. | | 0 | 1 | 2 | 3 | 4 |
| Jeg er tilfreds med den måde, jeg klarer min sygdom på …. | | 0 | 1 | 2 | 3 | 4 |
| Jeg er ved at give op i kampen mod min sygdom………… | | 0 | 1 | 2 | 3 | 4 |
| Jeg føler mig nervøs………………………………………. | | 0 | 1 | 2 | 3 | 4 |
| Jeg er bekymret for at dø…………………………………. | | 0 | 1 | 2 | 3 | 4 |
| Jeg er bekymret for, at min tilstand vil forværres………… | | 0 | 1 | 2 | 3 | 4 |
|  | |  |  |  |  |  |
| **FUNKTIONELT VELBEFINDENDE** | | **Slet**  **ikke** | **En lille smule** | **I nogen grad** | **En hel del** | **Meget** |
| Jeg er i stand til at arbejde (inkluderer arbejde i  hjemmet)…………………………………………………… | | 0 | 1 | 2 | 3 | 4 |
| Mit arbejde (inkluderer arbejde i hjemmet) er  tilfredsstillende……………………………………………… | | 0 | 1 | 2 | 3 | 4 |
| Jeg er i stand til at nyde livet………………………………. | | 0 | 1 | 2 | 3 | 4 |
| Jeg har accepteret min sygdom……………………………. | | 0 | 1 | 2 | 3 | 4 |
| Jeg sover godt………………………………………………... | | 0 | 1 | 2 | 3 | 4 |
| Jeg nyder det, jeg plejer at lave for min fornøjelses skyld… | | 0 | 1 | 2 | 3 | 4 |
| Lige nu er jeg tilfreds med min livskvalitet……………….. | | 0 | 1 | 2 | 3 | 4 |
|  | |  |  |  |  |  |

| **Ved at vælge ét tal i hver linje bedes du angive dit svar, sådan at det passer på de seneste 7 dage.** | | | | | | |
| --- | --- | --- | --- | --- | --- | --- |
| **ANDRE BEKYMRINGER** | | **Slet**  **ikke** | **En lille smule** | **I nogen grad** | **En hel del** | **Meget** |
|  | |  |  |  |  |  |
| Jeg føler mig udmattet……………………………………. | | 0 | 1 | 2 | 3 | 4 |
| Jeg føler mig svag i hele kroppen………………………… | | 0 | 1 | 2 | 3 | 4 |
| Jeg har mistet livslysten…………………………………... | | 0 | 1 | 2 | 3 | 4 |
| Jeg føler mig træt…………………………………………. | | 0 | 1 | 2 | 3 | 4 |
| Jeg har svært ved at komme i gang med noget, fordi jeg  er træt……………………………………………………... | | 0 | 1 | 2 | 3 | 4 |
| Jeg har svært ved at afslutte noget, fordi jeg er træt……… | | 0 | 1 | 2 | 3 | 4 |
| Jeg har energi……………………………………………... | | 0 | 1 | 2 | 3 | 4 |
| Jeg er i stand til at udføre mine normale aktiviteter………. | | 0 | 1 | 2 | 3 | 4 |
| Jeg har brug for at sove i løbet af dagen………………….. | | 0 | 1 | 2 | 3 | 4 |
| Jeg er for træt til at spise………………………………….. | | 0 | 1 | 2 | 3 | 4 |
| Jeg har brug for hjælp til at udføre mine normale  aktiviteter…………………………………………………. | | 0 | 1 | 2 | 3 | 4 |
| Jeg er frustreret over, at jeg er for træt til at gøre de ting,  jeg gerne vil………………………………………………. | | 0 | 1 | 2 | 3 | 4 |
| Jeg er nødt til at begrænse min selskabelige omgang  med andre, fordi jeg er træt………………………………. | | 0 | 1 | 2 | 3 | 4 |

| 1. Arbejde og beskæftigelse   Følgende spørgsmål omhandler, hvorledes din gigtsygdom indvirker på din evne til at arbejde og udføre almindelige aktiviteter. *Udfyld venligst de tomme felter eller sæt ring om et tal, som angivet.*  **C1 Er du ansat på nuværende tidspunkt (arbejder mod betaling)?** _____NEJ _____JA  *Hvis NEJ, afkryds “NEJ” og gå videre til spørgsmål C6*  De næste spørgsmål omhandler de **sidste syv dage**, men omfatter ikke dagen i dag.  **C2 I de sidste syv dage, hvor mange timer har du forsømt fra arbejdet på grund af problemer i forbindelse med din gigtsygdom?** *Medtag timer, du forsømte på sygedage, samt de gange, hvor du mødte sent, gik tidligt osv. på grund af* *din gigtsygdom. Medtag ikke den tid du forsømte for at deltage i denne undersøgelse.*  ______TIMER  **C3 I** **de sidste syv dage, hvor mange timer har du forsømt fra arbejdet af andre årsager som fx ferie og helligdage, eller tid, hvor du fik fri til at deltage i denne undersøgelse?**  ______TIMER  **C4 I de sidste syv dage, hvor mange timer arbejdede du rent faktisk?**  ______TIMER (*Hvis “0”, gå videre til spørgsmål C6)*  **C5 I de sidste syv dage, hvor meget påvirkede din gigtsygdom din produktivitet, mens du arbejdede?**   *Tænk på de dage, hvor du var begrænset i mængden eller typen af arbejde, som du kunne udføre, dage hvor du udrettede mindre end du gerne ville, eller dage hvor du ikke kunne udføre dit arbejde så omhyggeligt som sædvanligt. Hvis din gigtsygdom kun påvirkede dit arbejde lidt, skal du vælge et lavt tal. Vælg et højt tal, hvis din gigtsygdom påvirkede dit arbejde meget.*   \|  \|  \| \| \| \| \| \| \| \| \| \| \|  \| \| --- \| --- \| --- \| --- \| --- \| --- \| --- \| --- \| --- \| --- \| --- \| --- \| --- \| \|  \| Tænk kun på hvor meget din gigtsygdom påvirkede din produktivitet mens du arbejdede. \| \| \| \| \| \| \| \| \| \| \|  \| \| Min gigtsygdom  havde ingen indvirkning på \|  \|  \|  \|  \|  \|  \|  \|  \|  \|  \|  \| Min gigtsygdom forhindrede mig fuldstændig i \| \| mit arbejde \| 0 \| 1 \| 2 \| 3 \| 4 \| 5 \| 6 \| 7 \| 8 \| 9 \| 10 \| at arbejde \| \|  \| SÆT RING OM ET TAL \| \| \| \| \| \| \| \| \| \| \|  \|   **C6 I de sidste syv dage, hvor meget påvirkede din gigtsygdom din evne til at udføre almindelige daglige aktiviteter, ud over at passe et arbejde?**   *Med almindelige aktiviteter menes de sædvanlige aktiviteter, du udfører, som fx husarbejde, indkøb, børnepasning, motion, lektielæsning, osv. Tænk på de gange, hvor du var begrænset i mængden eller typen af aktiviteter, som du kunne udføre og de gange, hvor du udrettede mindre, end du gerne ville. Hvis din gigtsygdom kun påvirkede dine aktiviteter lidt, skal du vælge et lavt tal. Vælg et højt tal, hvis din gigtsygdom påvirkede dine aktiviteter meget.*     \| Tænk kun på hvor meget din gigtsygdom påvirkede din evne til at udføre almindelige daglige  aktiviteter, ud over at passe et arbejde. \| \| \| \| \| \| \| \| \| \| \| \| \| \| --- \| --- \| --- \| --- \| --- \| --- \| --- \| --- \| --- \| --- \| --- \| --- \| --- \| \|  \|  \| \| \| \| \| \| \| \| \| \| \|  \| \| Min gigtsygdom  havde ingen indvirkning på  mine daglige \|  \|  \|  \|  \|  \|  \|  \|  \|  \|  \|  \| Min gigtsygdom forhindrede mig fuldstændigt i  at udføre mine \| \| aktiviteter \| 0 \| 1 \| 2 \| 3 \| 4 \| 5 \| 6 \| 7 \| 8 \| 9 \| 10 \| daglige aktiviteter \| \|  \| SÆT RING OM ET TAL \| \| \| \| \| \| \| \| \| \| \|  \| |
| --- | --- | --- | --- | --- | --- | --- | --- | --- | --- | --- | --- | --- | --- | --- | --- | --- | --- | --- | --- | --- | --- | --- | --- | --- | --- | --- | --- | --- | --- | --- | --- | --- | --- | --- | --- | --- | --- | --- | --- | --- | --- | --- | --- | --- | --- | --- | --- | --- | --- | --- | --- | --- | --- | --- | --- | --- | --- | --- | --- | --- | --- | --- | --- | --- | --- | --- | --- | --- | --- | --- | --- | --- | --- | --- | --- | --- | --- | --- | --- | --- | --- | --- | --- | --- | --- | --- | --- | --- | --- | --- | --- | --- | --- | --- | --- | --- | --- | --- | --- | --- | --- | --- | --- | --- | --- | --- | --- | --- | --- | --- | --- | --- | --- | --- | --- | --- | --- | --- | --- | --- | --- | --- | --- | --- | --- | --- | --- | --- | --- | --- |

| 1. Livskvalitet   De følgende spørgsmål relaterer sig til din livskvalitet. Under hver overskrift bedes du sætte kryds i DEN kasse, der bedst beskriver dit helbred I DAG.   \| **BEVÆGELIGHED** \|  \| \| --- \| --- \| \| Jeg har ingen problemer med at gå omkring \| ❑ \| \| Jeg har lidt problemer med at gå omkring \| ❑ \| \| Jeg har moderate problemer med at gå omkring \| ❑ \| \| Jeg har store problemer med at gå omkring \| ❑ \| \| Jeg kan ikke gå omkring \| ❑ \| \| **PERSONLIG PLEJE** \|  \| \| Jeg har ingen problemer med at vaske mig eller klæde mig på \| ❑ \| \| Jeg har lidt problemer med at vaske mig eller klæde mig på \| ❑ \| \| Jeg har moderate problemer med at vaske mig eller klæde mig på \| ❑ \| \| Jeg har store problemer med at vaske mig eller klæde mig på \| ❑ \| \| Jeg kan ikke vaske mig eller klæde mig på \| ❑ \| \| **SÆDVANLIGE AKTIVITETER** *(fx. arbejde, studie, husarbejde, familie-*  *eller fritidsaktiviteter)* \|  \| \| Jeg har ingen problemer med at udføre mine sædvanlige aktiviteter \| ❑ \| \| Jeg har lidt problemer med at udføre mine sædvanlige aktiviteter \| ❑ \| \| Jeg har moderate problemer med at udføre mine sædvanlige aktiviteter \| ❑ \| \| Jeg har store problemer med at udføre mine sædvanlige aktiviteter \| ❑ \| \| Jeg kan ikke udføre mine sædvanlige aktiviteter \| ❑ \| \| **SMERTER / UBEHAG** \|  \| \| Jeg har ingen smerter eller ubehag \| ❑ \| \| Jeg har lidt smerter eller ubehag \| ❑ \| \| Jeg har moderate smerter eller ubehag \| ❑ \| \| Jeg har stærke smerter eller ubehag \| ❑ \| \| Jeg har ekstreme smerter eller ubehag \| ❑ \| \| **ANGST / DEPRESSION** \|  \| \| Jeg er ikke ængstelig eller deprimeret \| ❑ \| \| Jeg er lidt ængstelig eller deprimeret \| ❑ \| \| Jeg er moderat ængstelig eller deprimeret \| ❑ \| \| Jeg er meget ængstelig eller deprimeret \| ❑ \| \| Jeg er ekstremt ængstelig eller deprimeret \| ❑ \|      \| Vi vil gerne vide, hvor godt eller dårligt dit helbred er I DAG. \| \| --- \| \| Denne skala er nummereret fra 0 til 100. \| \| 100 svarer til det bedste helbred, du kan forestille dig. 0 svarer til det dårligste helbred, du kan forestille dig. \| \| Sæt et X på det sted på skalaen, der viser, hvordan dit helbred  er I DAG. \| \| Skriv derefter det tal, du har markeret på skalaen, ind i boksen nedenunder. \| |
| --- | --- | --- | --- | --- | --- | --- | --- | --- | --- | --- | --- | --- | --- | --- | --- | --- | --- | --- | --- | --- | --- | --- | --- | --- | --- | --- | --- | --- | --- | --- | --- | --- | --- | --- | --- | --- | --- | --- | --- | --- | --- | --- | --- | --- | --- | --- | --- | --- | --- | --- | --- | --- | --- | --- | --- | --- | --- | --- | --- | --- | --- | --- | --- | --- | --- |

Det bedste

helbred, du kan forestille dig

10

0

20

30

40

50

60

80

70

90

100

5

15

25

35

45

55

75

65

85

95

DIT HELBRED I DAG =

Det dårligste helbred, du kan forestille dig

1. Søvn

De næste spørgsmål handler om, hvordan du har sovet indenfor **de seneste 4** **uger.**

**E1 Hvor lang tid har du normalt været om at falde i søvn i de seneste 4 uger?***(Sæt ét kryds)*

❑ 0-15 minutter

❑ 16-30 minutter

❑ 31-45 minutter

❑ 46-60 minutter

❑ Mere end 60 minutter

**E2 Hvor mange timer har du i gennemsnit sovet hver nat i de seneste 4 uger?**

Skriv antal
timer pr. nat: __ __

**E3 Hvor ofte i de seneste 4 uger har du...**

|  | (*Sæt ét kryds på hver linje)* | | | | | |
| --- | --- | --- | --- | --- | --- | --- |
|  | **Hele tiden** | **Det meste af tiden** | **En stor del af tiden** | **Noget af tiden** | **En lille del af tiden** | **På intet tidspunkt** |
| følt, at din søvn ikke var  rolig (uro i kroppen,  anspændthed, talt i søvne  osv., mens du sov)? | 1 | 2 | 3 | 4 | 5 | 6 |
| fået så meget søvn, at du  følte dig udhvilet, når du  vågnede om morgenen? | 1 | 2 | 3 | 4 | 5 | 6 |
| vågnet stakåndet eller  med hovedpine? | 1 | 2 | 3 | 4 | 5 | 6 |
| følt dig døsig eller søvnig  i løbet af dagen? | 1 | 2 | 3 | 4 | 5 | 6 |
| haft svært ved at falde i  søvn? | 1 | 2 | 3 | 4 | 5 | 6 |
| vågnet og haft svært ved  at falde i søvn igen? | 1 | 2 | 3 | 4 | 5 | 6 |
| haft svært ved at holde  dig vågen i løbet af  dagen? | 1 | 2 | 3 | 4 | 5 | 6 |
| snorket, når du har sovet? | 1 | 2 | 3 | 4 | 5 | 6 |
| taget en lur eller flere (5  minutter eller mere) i  løbet af dagen? | 1 | 2 | 3 | 4 | 5 | 6 |
| fået så mange timers  søvn, som du havde brug for? | 1 | 2 | 3 | 4 | 5 | 6 |

1. Psykisk velbefindende

**De følgende spørgsmål går på, hvordan du har haft det gennem de sidste 2 uger*.*Besvar venligst spørgsmålene ved at sætte et kryds ved det tal der svarer til, hvorledes du har følt det. Bemærk at et højere tal betyder mere depression.**

| **Hvor meget af tiden inden for de seneste 14 dage** | **Hele**  **tiden** | **Det meste af tiden** | **Lidt over halvdelen af tiden** | **Lidt under halvdelen af tiden** | **Lidt af**  **tiden** | **På intet tidspunkt** |
| --- | --- | --- | --- | --- | --- | --- |
| Har du følt dig trist til mode, ked af det? | 5□ | 4□ | 3□ | 2□ | 1□ | 0□ |
| Har du manglet interesse for dine daglige gøremål? | 5□ | 4□ | 3□ | 2□ | 1□ | 0□ |
| Har du følt, at du manglede energi og kræfter? | 5□ | 4□ | 3□ | 2□ | 1□ | 0□ |
| Har du haft mindre selvtillid? | 5□ | 4□ | 3□ | 2□ | 1□ | 0□ |
| Har du haft dårlig samvittighed eller skyldfølelse? | 5□ | 4□ | 3□ | 2□ | 1□ | 0□ |
| Har du følt, at livet ikke var værd at leve? | 5□ | 4□ | 3□ | 2□ | 1□ | 0□ |
| Har du haft besvær med at  koncentrere dig, fx at læse avis eller følge med i fjernsyn? | 5□ | 4□ | 3□ | 2□ | 1□ | 0□ |
| Har du følt dig rastløst? | 5□ | 4□ | 3□ | 2□ | 1□ | 0□ |
| Har du følt dig mere stille? | 5□ | 4□ | 3□ | 2□ | 1□ | 0□ |
| Har du sovet for lidt? | 5□ | 4□ | 3□ | 2□ | 1□ | 0□ |
| Har du sovet for meget? | 5□ | 4□ | 3□ | 2□ | 1□ | 0□ |
| Har du haft nedsat appetit? | 5□ | 4□ | 3□ | 2□ | 1□ | 0□ |
| Har du haft øget appetit? | 5□ | 4□ | 3□ | 2□ | 1□ | 0□ |

G. Daglige gøremål

**G1 De følgende spørgsmål drejer sig om, hvordan du klarer dig i det daglige. Sæt kryds ved det svar, som bedst beskriver, hvordan du klarer dig for tiden. Hvis du bruger hjælpemidler, så skal du svare på, hvordan du klarer dig med hjælpemidlerne.**

*Sæt kun ét kryds ud for hvert spørgsmål.*

|  | Ja, uden besvær | Ja, med  noget besvær | Ja, med meget besvær | Nej, det kan jeg ikke |
| --- | --- | --- | --- | --- |
| Kan du selv klæde dig på?  (det gælder også snørebånd og knapper) | □ | □ | □ | □ |
| Kan du selv vaske dit hår? | □ | □ | □ | □ |
| Kan du rejse dig fra en spisestuestol? | □ | □ | □ | □ |
| Kan du selv klare at komme i og ud af en seng? | □ | □ | □ | □ |
| Kan du selv skære et stykke stegt kød i stykker? | □ | □ | □ | □ |
| Kan du løfte en fyldt kop eller et fyldt glas? | □ | □ | □ | □ |
| Kan du selv åbne en ny mælkekarton? | □ | □ | □ | □ |
| Kan du selv gå rundt udendørs, hvor der er fladt? | □ | □ | □ | □ |
| Kan du selv gå 5 trin op ad en trappe? | □ | □ | □ | □ |
| Kan du selv vaske og tørre dig over det hele? | □ | □ | □ | □ |
| Kan du selv tage karbad? | □ | □ | □ | □ |
| Kan du selv klare toiletbesøg? | □ | □ | □ | □ |
|  |  |  |  |  |
|  |  |  |  |  |
|  |  |  |  |  |
|  | Ja, uden besvær | Ja, med  noget besvær | Ja, med meget besvær | Nej, det kan jeg ikke |
| Kan du nå op, og hente noget tungt ned fra en hylde over hovedhøjde (fx 2 kg sukker)? | □ | □ | □ | □ |
| Kan du selv samle f.eks. tøj op fra gulvet? | □ | □ | □ | □ |
| Kan du selv åbne en bildør? | □ | □ | □ | □ |
| Kan du selv skrue låget af et glas, der har været åbnet før? | □ | □ | □ | □ |
| Kan du åbne og lukke en almindelig vandhane? | □ | □ | □ | □ |
| Kan du selv klare indkøb og andre ærinder? | □ | □ | □ | □ |
| Kan du selv komme ind og ud af en bil? | □ | □ | □ | □ |
| Kan du selv klare husarbejdet fx støvsugning  eller lettere havearbejde? | □ | □ | □ | □ |

**G2 Hvis du bruger hjælpemidler, så sæt kryds ved alle de hjælpemidler, som du bruger.**

❑ Hjælpemidler til påklædning (til knapper, strømper og lynlåse og lign.)

❑ Speciel eller forhøjet stol

❑ Specielle køkkenredskaber

❑ Specielle spiseredskaber

❑ Almindelig stok

❑ Krykkestok(ke)

❑ Gangstativ eller rollator

❑ Kørestol

❑ Forhøjet toiletsæde

❑ Badestol

❑ Langskaftede hjælpemidler til badeværelset

❑ Håndtag i badeværelse eller på toilettet

❑ Langskaftede hjælpemidler for at nå ting

❑ Tang til skruelåg

❑ Bruger du andre hjælpemidler? Skriv venligst hvilke: ________________________________________________________________________________________________________________________________________________________________________________________________________________________________________________

Du bedes besvare de følgende tre spørgsmål ved at sætte en kort streg gennem linien på det punkt, som du mener passer til svaret på spørgsmålet.

| Forkert | Rigtigt |
| --- | --- |
| 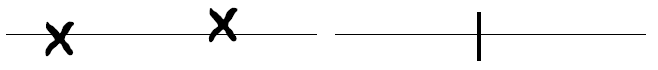 | |

**G3 Hvor mange gigtsmerter har du for tiden?**

| Ingen |  |  |  |  |  |  |  |  |  |  | Uudholdelige |
| --- | --- | --- | --- | --- | --- | --- | --- | --- | --- | --- | --- |
| gigtsmerter |  |  |  |  |  |  |  |  |  |  | gigtsmerter |

**G4 Hvor træt er du for tiden?**

| Slet |  |  |  |  |  |  |  |  |  |  | Uudholdeligt |
| --- | --- | --- | --- | --- | --- | --- | --- | --- | --- | --- | --- |
| ikke |  |  |  |  |  |  |  |  |  |  | meget |

**G5. Hvor meget påvirker gigten som helhed din tilværelse for tiden?**

| Slet |  |  |  |  |  |  |  |  |  |  | Uudholdeligt |
| --- | --- | --- | --- | --- | --- | --- | --- | --- | --- | --- | --- |
| ikke |  |  |  |  |  |  |  |  |  |  | meget |

| Mange tak for din deltagelse i vores undersøgelse. Vi er meget glade for, at du ville bruge tiden på det. |
| --- |
