# Supplementary material for: Severity of fatigue in people with rheumatoid arthritis, psoriatic arthritis and spondyloarthritis – Results of a cross-sectional study
Source: PLoS One. 2019 Jun 28;14(6):e0218831. doi: 10.1371/journal.pone.0218831 (PMC6599141; doi:10.1371/journal.pone.0218831)
Supplement: S4 File — (DOCX) [file pone.0218831.s004.docx]

# Thank you for participating

in the study on arthritis and fatigue, which we carry out at the Centre for Rheumatology and Spine Diseases in the fall/winter of 2017.

| Welcome to the questionnaire We are pleased that you want to participate.  The questionnaire contains questions about how you are doing in everyday life with your rheumatic disease, including pain, fatigue and sleep, experienced quality of life as well as work ability. You may find that some questions are similar to each other. It is important that you answer all the questions anyway.  It takes approximately 20 minutes to answer the questionnaire. All information collected in connection with the questionnaire will be treated confidentially. |
| --- |

Write the four-digit serial number

___ ___ ___ ___

| 1. Background and education   The first questions in the questionnaire relate to your background and education. |
| --- |

A1 In which year were you born?
*Write the year with four numbers: YYYY*

___ ___ ___ ___

**A2 How many years ago were you diagnosed with arthritis?**
*Tick one box only*

❑ 0-5 years

❑ 6-10 years

❑ 11-15 years

❑ 16-20 years

❑ More than 20 years

A3 What type of household do you live in?
*Tick one box only*

❑ Married/living together with children

❑ Married/living together without children

❑ Single with children

❑ Single without children

❑ Living at home with parents

❑ Shared living with other adults

A4 What is your highest level of education?
*Tick one box only*

❑ Elementary school

❑ High school

❑ Secondary school

❑ Short-cycle tertiary

❑ Bachelor’s degree

❑ Master’s degree

**A5 What is your current employment situation (main occupation)?**

*Tick one box only*

❑ Employed full-time

❑ Employed part-time

❑ Subsidised job

❑ Student

❑ Homemaker

❑ Unemployed

❑ Age-related retirement

❑ Health-related retirement because of arthritis

❑ Health-related retirement for other reasons

❑ Other. Please specify:

**A6 What is your household’s annual income before tax?**
*Tick one box only*

❑ Less than 200,000 DKK

❑ 200,000 DKK-399,999 DKK

❑ 400,000 DKK-599,999 DKK

❑ 600,000 DKK or more

❑ Prefer not to say

| 1. Well-being   The questions on the next pages concerns your physical, social, emotional and functional well-being. The questions are formulated as statements. For each statement, please circle the number that best matches how you have felt in the past 7 days. |
| --- |

| Below is a list of statements that other people with your illness have said are important. **Please circle**  **or mark one number per line to indicate your response as it applies to the past 7 days.** | | | | | | |
| --- | --- | --- | --- | --- | --- | --- |
| **PHYSICAL WELL-BEING** | | **Not at all** | **A little bit** | **Some-what** | **Quite a bit** | **Very much** |
|  | |  |  |  |  |  |
| I have lack of energy……………………………................. | | 0 | 1 | 2 | 3 | 4 |
| I have nausea..…………………………………………….. | | 0 | 1 | 2 | 3 | 4 |
| Because of my physical condition, I have trouble meeting  the needs of my family.…………………………………… | | 0 | 1 | 2 | 3 | 4 |
| I have pain…..…………………………………………….. | | 0 | 1 | 2 | 3 | 4 |
| I am bothered by side effects of treatment…..……………. | | 0 | 1 | 2 | 3 | 4 |
| I feel ill…………………………………………….............. | | 0 | 1 | 2 | 3 | 4 |
| I am forced to spend time in bed…………………………... | | 0 | 1 | 2 | 3 | 4 |
|  | |  |  |  |  |  |
| **SOCIAL/FAMILY WELL-BEING** | | **Not at all** | **A little bit** | **Some-what** | **Quite a bit** | **Very much** |
|  | |  |  |  |  |  |
| I feel close to friends…………………………...….............. | | 0 | 1 | 2 | 3 | 4 |
| I get emotional support from my family..…………………. | | 0 | 1 | 2 | 3 | 4 |
| I get support from my friends.…………………………….. | | 0 | 1 | 2 | 3 | 4 |
| My family has accepted my illness…………………….… | | 0 | 1 | 2 | 3 | 4 |
| I am satisfied with family communication about my illness…………………………..………………………… | | 0 | 1 | 2 | 3 | 4 |
| I feel close to my partner (or the person who is my main support)………………..………....……………………….. | | 0 | 1 | 2 | 3 | 4 |
| Regardless of your current level of sexual activity, please answer the following question. If you prefer not to answer it, please mark this box and go to the next section. | |  |  |  |  |  |
| I am satisfied with my sex life..…………………………... | | 0 | 1 | 2 | 3 | 4 |

| **Please circle or mark one number per line to indicate your response as it applies to the past 7 days.** | | | | | |
| --- | --- | --- | --- | --- | --- |
| **EMOTIONAL WELL-BEING** | **Not at all** | **A little bit** | **Some-what** | **Quite a bit** | **Very much** |
|  |  |  |  |  |  |
| I feel sad……..……………………………………………. | 0 | 1 | 2 | 3 | 4 |
| I am satisfied with how I am coping with my illness…..…. | 0 | 1 | 2 | 3 | 4 |
| I am losing hope in the fight against my illness…………… | 0 | 1 | 2 | 3 | 4 |
| I feel nervous………………………………………………. | 0 | 1 | 2 | 3 | 4 |
| I worry about dying…….…………………………………. | 0 | 1 | 2 | 3 | 4 |
| I worry about my condition will get worse……..………… | 0 | 1 | 2 | 3 | 4 |
|  |  |  |  |  |  |
| **FUNCTIONAL WELL-BEING** | **Not at all** | **A little bit** | **Some-what** | **Quite a bit** | **Very much** |
| I am able to work (include work at home)…………………… | 0 | 1 | 2 | 3 | 4 |
| My work (include work at home) is fulfilling……………… | 0 | 1 | 2 | 3 | 4 |
| I am able to enjoy life…….………………………………. | 0 | 1 | 2 | 3 | 4 |
| I have accepted my illness…………………………………. | 0 | 1 | 2 | 3 | 4 |
| I am sleeping well.………………………………………... | 0 | 1 | 2 | 3 | 4 |
| I am enjoying the times I usually do for fun……………… | 0 | 1 | 2 | 3 | 4 |
| I am content with the quality of my life right now………….. | 0 | 1 | 2 | 3 | 4 |

| **Please circle or mark one number per line to indicate your response as it applies to the past 7 days.** | | | | | | |
| --- | --- | --- | --- | --- | --- | --- |
| **ADDITIONAL CONCERNS** | | **Not at all** | **A little bit** | **Some-what** | **Quite a bit** | **Very much** |
|  | |  |  |  |  |  |
| I feel fatigued………..……………………………………. | | 0 | 1 | 2 | 3 | 4 |
| I feel weak all over………………………………………… | | 0 | 1 | 2 | 3 | 4 |
| I feel listless (“washed out”)………………………………... | | 0 | 1 | 2 | 3 | 4 |
| I feel tired…………………………………………………. | | 0 | 1 | 2 | 3 | 4 |
| I have trouble starting things because I am tired…………... | | 0 | 1 | 2 | 3 | 4 |
| I have trouble finishing things because I am tired………… | | 0 | 1 | 2 | 3 | 4 |
| I have energy.……………………………………………... | | 0 | 1 | 2 | 3 | 4 |
| I am able to do my usual activities………………..………. | | 0 | 1 | 2 | 3 | 4 |
| I need sleep during the day…………….………………….. | | 0 | 1 | 2 | 3 | 4 |
| I am too tired to eat……..………………………………….. | | 0 | 1 | 2 | 3 | 4 |
| I need help doing my usual activities...……………………. | | 0 | 1 | 2 | 3 | 4 |
| I am frustrated by being too tired to do the things I want  to do……….………………………………………………. | | 0 | 1 | 2 | 3 | 4 |
| I have to limit my social activity because I am tired………. | | 0 | 1 | 2 | 3 | 4 |

| 1. Work and employment   The following questions ask about the effect of your arthritis on your ability to work and perform regular activities. *Please fill in the blanks or circle a number, as indicated.*  **C1 Are you currently employed (working for pay)?** _____NO _____YES  *If NO, check ”NO” and skip to question C6*  The next questions are about the **past seven days**, not including today.  **C2 During the past seven days, how many hours did you miss from work because of problems associated with your arthritis?** *Include hours you missed on sick days, times you went in late, left early, etc., because of your arthritis. Do not include time you missed to participate in this study.*  ______HOURS  **C3 During the past seven days, how many hours did you miss from work because of any other reason, such as vacation, holidays, time off to participate in this study?**  ______HOURS  **C4 During the past seven days, how many hours did you actually work?**  ______HOURS (*If ”0”, skip to question C6)*  **C5 During the past seven days, how much did your arthritis affect your productivity while you were working?**   *Think about days you were limited in the amount or kind of work you could do, days you accomplished less than you would like, or days you could not do your work as carefully as usual. If arthritis affected your work only a little, choose a low number. Choose a high number if arthritis affected your work a great deal.*   \|  \|  \| \| \| \| \| \| \| \| \| \| \|  \| \| --- \| --- \| --- \| --- \| --- \| --- \| --- \| --- \| --- \| --- \| --- \| --- \| --- \| \|  \| Consider only how much arthritis affected productivity while you were working. \| \| \| \| \| \| \| \| \| \| \|  \| \| Arthritis had no effect on my \|  \|  \|  \|  \|  \|  \|  \|  \|  \|  \|  \| Arthritis completely \| \| work \| 0 \| 1 \| 2 \| 3 \| 4 \| 5 \| 6 \| 7 \| 8 \| 9 \| 10 \| prevented me  from working \| \|  \| CIRCLE A NUMBER \| \| \| \| \| \| \| \| \| \| \|  \|   **C6 During the past seven days, how much did your arthritis affect your ability to do your regular daily activities, other than work at a job?**  *By regular activities, we mean the usual activities you do, such as work around the house, shopping, childcare, exercising, studying, etc. Think about times you were limited in the amount or kind of activities you could do and times you accomplished less than you would like. If arthritis affected your activities only a little, choose a low number. Choose a high number if arthritis affected your activities a great deal.*     \| Consider only how much arthritis affected your ability  to do your regular daily activities, other than work at a job. \| \| \| \| \| \| \| \| \| \| \| \| \| \| --- \| --- \| --- \| --- \| --- \| --- \| --- \| --- \| --- \| --- \| --- \| --- \| --- \| \|  \|  \| \| \| \| \| \| \| \| \| \| \|  \| \| Arthritis had  no effect on my \|  \|  \|  \|  \|  \|  \|  \|  \|  \|  \|  \| Arthritis  completely \| \| daily activities \| 0 \| 1 \| 2 \| 3 \| 4 \| 5 \| 6 \| 7 \| 8 \| 9 \| 10 \| prevented me from doing my  daily activities \| \|  \| CIRCLE A NUMBER \| \| \| \| \| \| \| \| \| \| \|  \| |
| --- | --- | --- | --- | --- | --- | --- | --- | --- | --- | --- | --- | --- | --- | --- | --- | --- | --- | --- | --- | --- | --- | --- | --- | --- | --- | --- | --- | --- | --- | --- | --- | --- | --- | --- | --- | --- | --- | --- | --- | --- | --- | --- | --- | --- | --- | --- | --- | --- | --- | --- | --- | --- | --- | --- | --- | --- | --- | --- | --- | --- | --- | --- | --- | --- | --- | --- | --- | --- | --- | --- | --- | --- | --- | --- | --- | --- | --- | --- | --- | --- | --- | --- | --- | --- | --- | --- | --- | --- | --- | --- | --- | --- | --- | --- | --- | --- | --- | --- | --- | --- | --- | --- | --- | --- | --- | --- | --- | --- | --- | --- | --- | --- | --- | --- | --- | --- | --- | --- | --- | --- | --- | --- | --- | --- | --- | --- | --- | --- | --- | --- |

| 1. Quality of life   Under each heading, please tick the ONE box that best describes your health TODAY.   \| **MOBILITY** \|  \| \| --- \| --- \| \| I have no problems in walking about \| ❑ \| \| I have slight problems walking about \| ❑ \| \| I have moderate problems in walking about \| ❑ \| \| I have severe problems in walking about \| ❑ \| \| I am unable to walk about \| ❑ \| \| **SELF-CARE** \|  \| \| I have no problems washing or dressing myself \| ❑ \| \| I have slight problems washing or dressing myself \| ❑ \| \| I have moderate problems washing or dressing myself \| ❑ \| \| I have severe problems washing or dressing myself \| ❑ \| \| I am unable to wash or dress myself \| ❑ \| \| **USUAL ACTIVITIVES** *(e.g. work, study, housework, family or  leisure activities)* \|  \| \| I have no problems doing my usual activities \| ❑ \| \| I have slight problems doing my usual activities \| ❑ \| \| I have moderate problems doing my usual activities \| ❑ \| \| I have severe problems doing my usual activities \| ❑ \| \| I am unable to wash or dress myself \| ❑ \| \| **PAIN/DISCOMFORT** \|  \| \| I have no pain or discomfort \| ❑ \| \| I have slight pain or discomfort \| ❑ \| \| I have moderate pain or discomfort \| ❑ \| \| I have severe pain or discomfort \| ❑ \| \| I have extreme pain or discomfort \| ❑ \| \| **ANXIETY/DEPRESSION** \|  \| \| I am not anxious or depressed \| ❑ \| \| I am slightly anxious or depressed \| ❑ \| \| I am moderately anxious or depressed \| ❑ \| \| I am severely anxious or depressed \| ❑ \| \| I am extremely anxious or depressed \| ❑ \|      \| We would like to know how good or bad your health is TODAY. \| \| --- \| \| This scale is numbered from 0 to 100. \| \| 100 means the best health you can imagine. 0 means the worst health you can imagine. \| \| Mark an X on the scale to indicate how your health is TODAY. \| \| Now, please write the number you marked on the scale in the box below. \| |
| --- | --- | --- | --- | --- | --- | --- | --- | --- | --- | --- | --- | --- | --- | --- | --- | --- | --- | --- | --- | --- | --- | --- | --- | --- | --- | --- | --- | --- | --- | --- | --- | --- | --- | --- | --- | --- | --- | --- | --- | --- | --- | --- | --- | --- | --- | --- | --- | --- | --- | --- | --- | --- | --- | --- | --- | --- | --- | --- | --- | --- | --- | --- | --- | --- | --- |

The best health
you can imagine

10

0

20

30

40

50

60

80

70

90

100

5

15

25

35

45

55

75

65

85

95

YOUR HEALTH TODAY =

The worst health
you can imagine

1. Sleep

The next question is about now you slept within **the past 4 weeks.**

**E1 How long did it usually take for you to fall asleep during the past 4 weeks?***(Circle One)*

❑ 0-15 minutes

❑ 16-30 minutes

❑ 31-45 minutes

❑ 46-60 minutes

❑ More than 60 minutes

**E2 On the average, how many hours did you sleep each night during the past 4 weeks?**

Write in number
of hours per night: __ __

**E3 How often during the past 4 weeks did you...**

|  | (*Circle One Number On Each Line)* | | | | | |
| --- | --- | --- | --- | --- | --- | --- |
|  | **All of  the  Time** | **Most  of the  Time** | **A Good  Bit of  the Time** | **Some  of the  Time** | **A Little  of the  Time** | **None  of the  Time** |
| feel that your sleep was not quiet (moving restlessly, feeling tense, speaking, etc., while sleeping)? | 1 | 2 | 3 | 4 | 5 | 6 |
| get enough sleep to feel rested upon waking in the morning? | 1 | 2 | 3 | 4 | 5 | 6 |
| awaken short of breath or with a headache? | 1 | 2 | 3 | 4 | 5 | 6 |
| feel drowsy or sleepy during the day? | 1 | 2 | 3 | 4 | 5 | 6 |
| have trouble falling asleep? | 1 | 2 | 3 | 4 | 5 | 6 |
| awaken during your sleep time and have trouble falling asleep again? | 1 | 2 | 3 | 4 | 5 | 6 |
| have trouble staying awake during the day? | 1 | 2 | 3 | 4 | 5 | 6 |
| snore during your sleep? | 1 | 2 | 3 | 4 | 5 | 6 |
| take naps (5 minutes or longer) during the day? | 1 | 2 | 3 | 4 | 5 | 6 |
| get the amount of sleep you needed? | 1 | 2 | 3 | 4 | 5 | 6 |

1. Mental well-being

**The following questions ask about how you have been feeling over the last two weeks. Please put a tick in the box which is closest to how you have been feeling. A higher number signifies a higher degree of depression.**

| **How much of the time in the last two weeks** | **All the time** | **Most of  the time** | **Slightly more than half the time** | **Slightly less than half the time** | **Some of the  time** | **At no time** |
| --- | --- | --- | --- | --- | --- | --- |
| Have you felt low in spirits or sad? | 5□ | 4□ | 3□ | 2□ | 1□ | 0□ |
| Have you lost interest in your daily activities? | 5□ | 4□ | 3□ | 2□ | 1□ | 0□ |
| Have you felt lacking in energy and strength? | 5□ | 4□ | 3□ | 2□ | 1□ | 0□ |
| Have you felt less self-confident? | 5□ | 4□ | 3□ | 2□ | 1□ | 0□ |
| Have you had bad conscience or feelings of guilt? | 5□ | 4□ | 3□ | 2□ | 1□ | 0□ |
| Have you felt that life wasn’t worth living? | 5□ | 4□ | 3□ | 2□ | 1□ | 0□ |
| Have you had difficulty in concentrating, e.g. when reading the newspaper or watching TV? | 5□ | 4□ | 3□ | 2□ | 1□ | 0□ |
| Have you felt very restless? | 5□ | 4□ | 3□ | 2□ | 1□ | 0□ |
| Have you felt subdued or slowed down? | 5□ | 4□ | 3□ | 2□ | 1□ | 0□ |
| Have you been sleeping too little? | 5□ | 4□ | 3□ | 2□ | 1□ | 0□ |
| Have you been sleeping too much? | 5□ | 4□ | 3□ | 2□ | 1□ | 0□ |
| Have you suffered from reduced appetite? | 5□ | 4□ | 3□ | 2□ | 1□ | 0□ |
| Have you suffered from increased appetite? | 5□ | 4□ | 3□ | 2□ | 1□ | 0□ |

G. Daily activities

**G1 The following questions concern how you manage everyday activities. Tick the box with the answer that best describes how your abilities are at the moment. If you use aids or devices you have to answer how your abilities are when using the aids or devices.**

*Tick one box for each question.*

|  | Without any difficulty | With some difficulty | With much difficulty | Unable  to do |
| --- | --- | --- | --- | --- |
| Are you able to dress yourself, including shoelaces and buttons? | □ | □ | □ | □ |
| Are you able to shampoo your hair? | □ | □ | □ | □ |
| Are you able to stand up from a straight chair? | □ | □ | □ | □ |
| Are you able to get in and out of bed? | □ | □ | □ | □ |
| Are you able to cut your own meat? | □ | □ | □ | □ |
| Are you able to lift a full cup or glass to your mouth? | □ | □ | □ | □ |
| Are you able to open a new milk carton? | □ | □ | □ | □ |
| Are you able to walk outdoors on flat ground? | □ | □ | □ | □ |
| Are you able to climb up five steps? | □ | □ | □ | □ |
| Are you able to wash and dry your body? | □ | □ | □ | □ |
| Take a tub bath? | □ | □ | □ | □ |
| Get on and off the toilet? | □ | □ | □ | □ |
|  |  |  |  |  |
|  |  |  |  |  |
|  |  |  |  |  |
|  | Without any difficulty | With some difficulty | With much difficulty | Unable  to do |
| Are you able to reach and get down a 5 pound object (such as a bag of sugar) from above your head? | □ | □ | □ | □ |
| Are you able to bend down to pick up clothing from the floor? | □ | □ | □ | □ |
| Are you able to open car doors? | □ | □ | □ | □ |
| Are you able to open previously opened jars? | □ | □ | □ | □ |
| Are you able to turn faucets on and off? | □ | □ | □ | □ |
| Are you able to run errand and shop? | □ | □ | □ | □ |
| Are you able to get in and out of a car? | □ | □ | □ | □ |
| Are you able to do chores such as vacuuming or yard work? | □ | □ | □ | □ |

**G2 If you use aids or devices, tick all the aids and devices that you use.**

❑ Devices used for dressing (button hook, zipper pull, etc.)

❑ Special or built up chair

❑ Special kitchen utensils

❑ Special eating utensils

❑ Cane

❑ Crutches

❑ Walker

❑ Wheelchair

❑ Raised toilet seat

❑ Bathtub seat

❑ Long-handled appliances in bathroom

❑ Handle in bathroom or lavatory

❑ Long-handled appliances for reach

❑ Jar opener (for jars previously opened)

❑ Do you use other aids or devices? Please specify: ________________________________________________________________________________________________________________________________________________________________________________________________________________________________________________

Please answer the following three questions by drawing a short line through the line at the point that you think fits the answer to the question.

| Wrong | Right |
| --- | --- |
| 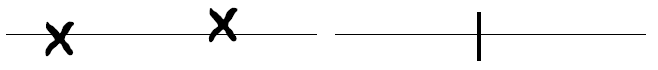 | |

**G3 How much arthritis pain do you have these days?**

| No |  |  |  |  |  |  |  |  |  |  | Unbearable |
| --- | --- | --- | --- | --- | --- | --- | --- | --- | --- | --- | --- |
| arthritis pain |  |  |  |  |  |  |  |  |  |  | arthritis pain |

**G4 How tired are you these days?**

| Not at |  |  |  |  |  |  |  |  |  |  | Unbearably |
| --- | --- | --- | --- | --- | --- | --- | --- | --- | --- | --- | --- |
| all |  |  |  |  |  |  |  |  |  |  | much |

**G5. How much does the arthritis in general affect your life these days?**

| Not at |  |  |  |  |  |  |  |  |  |  | Unbearably |
| --- | --- | --- | --- | --- | --- | --- | --- | --- | --- | --- | --- |
| all |  |  |  |  |  |  |  |  |  |  | much |

| Thank you very much for participating in our study. We are very pleased that you took the time to complete the questionnaire. |
| --- |
